# Supplementary material for: Uptake of complete postnatal care services and its determinants among rural women in Southern Ethiopia: Community-based cross-sectional study based on the current WHO recommendation
Source: PLoS One. 2021 Feb 3;16(2):e0246243. doi: 10.1371/journal.pone.0246243 (PMC7857562; doi:10.1371/journal.pone.0246243)
Supplement: S1 Questionnaire — (DOCX) [file pone.0246243.s002.docx]

| Results of questionnaire: - | |
| --- | --- |
| 1. Completed [_____] | 1. Participant Refused [_____] |
| 1. Partially completed [_____] | **4.** |

| Respondents identification ______  Questionnaire Code______  **Instruction: Circle the appropriate answer** provided and where applicable writes the required responses in the spaces provided.  **SECTION 1: socio-demographic and economic characteristics of respondents** | | | | | | | | | | | | | | | | | | | | | | | | | | | | | | | | | | | |
| --- | --- | --- | --- | --- | --- | --- | --- | --- | --- | --- | --- | --- | --- | --- | --- | --- | --- | --- | --- | --- | --- | --- | --- | --- | --- | --- | --- | --- | --- | --- | --- | --- | --- | --- | --- |
| S.N | Questions | | | | | | Answers | | | | | | | | | | | | | | | | | | | | | | | | | | | Code | |
| 101 | How old are you? Age (in years) | | | | | | 1. [___________]  2. I don’t know------------ | | | | | | | | | | | | | | | | | | | | | | | | | | |  | |
| 102 | What is your Marital status? | | | | | | 1. Married | | | | | | | | | | | | | | | | | 1. divorced /Separated | | | | | | | | | |  | |
|  |  |  |  |  |  |  | 1. un married | | | | | | | | | | | | | | | | | 1. Widowed | | | | | | | | | |  |  |
| 103 | What is your religion? | | | | | | 1. Orthodox | | | | | | | | | | | | | | | | | 1. Muslim | | | | | | | | | |  | |
|  |  |  |  |  |  |  | 1. Catholic | | | | | | | | | | | | | | | | | 1. Protestant | | | | | | | | | |  |  |
|  |  |  |  |  |  |  | 1. Other | | | | | | | | | | | | | | | | |  | | | | | | | | | |  |  |
| 104 | To what ethnicity you belong to? | | | | | | 1. Guraghe 2. Amhara  3. Oromo 4. Others. | | | | | | | | | | | | | | | | | | | | | | | | | | |  | |
| 105 | What is your educational level? | | | | | | 1. No formal education | | | | | | | | | | | | | | 1. 1-8^th^ | | | | | | | | | | | | |  | |
|  |  |  |  |  |  |  | 1. 9-12^th^ | | | | | | | | | | | | | | 1. College and above | | | | | | | | | | | | |  |  |
| 106 | What is your present Occupation? | | | | | | 1. House wife | | | | | | | | | | | | | | 1. Merchant | | | | | | | | | | | | |  | |
|  |  |  |  |  |  |  | 1. Farmer | | | | | | | | | | | | | | 1. Daily laborer | | | | | | | | | | | | |  |  |
|  |  |  |  |  |  |  | 1. civil servant | | | | | | | | | | | | | | 1. Others | | | | | | | | | | | | |  |  |
| 107 | What is the educational level of your husband? | | | | | | 1. No formal education | | | | | | | | | | | | | | 1. 1-8^th^ | | | | | | | | | | | | |  | |
|  |  |  |  |  |  |  | 1. 9-12^th^ | | | | | | | | | | | | | | 1. College and above | | | | | | | | | | | | |  |  |
| 108 | What is his present Occupation? | | | | | | 1. farmer | | | | | | | | | | | | | | 2.Government employee | | | | | | | | | | | | |  | |
|  |  |  |  |  |  |  | 3. Merchant | | | | | | | | | | | | | | 4. Daily work | | | | | | | | | | | | |  |  |
|  |  |  |  |  |  |  | 5. other | | | | | | | | | | | | | | 6. No work | | | | | | | | | | | | |  |  |
| 109 | How many members are there within the family?(family size) | | | | | | [________________] | | | | | | | | | | | | | | | | | | | | | | | | | | |  | |
| 110. | Wealth index measurement | | | | | | | | | | | | |  | | | | | | | | | | | | | | | | | | | | Code | |
| 1 | Does your household have:(1=Yes, 0=No) | | | | | | | | | | | | | Electricity | | | | | | | | | | | | | | | | | | | |  | |
|  |  |  |  |  |  |  |  |  |  |  |  |  |  | Radio | | | | | | | | | | | | | | | | | | | |  | |
|  |  |  |  |  |  |  |  |  |  |  |  |  |  | Television | | | | | | | | | | | | | | | | | | | |  | |
|  |  |  |  |  |  |  |  |  |  |  |  |  |  | mobile telephone | | | | | | | | | | | | | | | | | | | |  | |
|  |  |  |  |  |  |  |  |  |  |  |  |  |  | A table | | | | | | | | | | | | | | | | | | | |  | |
|  |  |  |  |  |  |  |  |  |  |  |  |  |  | A chair | | | | | | | | | | | | | | | | | | | |  | |
|  |  |  |  |  |  |  |  |  |  |  |  |  |  | Bed | | | | | | | | | | | | | | | | | | | |  | |
| 2 | Does anyone of your household member have? (1=Yes, 0=No) | | | | | | | | | | | | | Bicycle | | | | | | | | | | | | | | | | | | | |  | |
|  |  |  |  |  |  |  |  |  |  |  |  |  |  | Motorcycle | | | | | | | | | | | | | | | | | | | |  | |
|  |  |  |  |  |  |  |  |  |  |  |  |  |  | Bajaj | | | | | | | | | | | | | | | | | | | |  | |
|  |  |  |  |  |  |  |  |  |  |  |  |  |  | An animal-drawn cart? | | | | | | | | | | | | | | | | | | | |  | |
|  |  |  |  |  |  |  |  |  |  |  |  |  |  | A car/truck? | | | | | | | | | | | | | | | | | | | |  | |
| 3 | Do you have private home?(1=Yes, 0=No) | | | | | | | | | | | | |  | | | | | | | | | | | | | | | | | | | |  | |
| 4 | Main material of the roof (observe) | | | | | | | | | | | | | 1. Grass 2. Metal/corrugated iron | | | | | | | | | | | | | | | | | | | |  | |
| 5 | What is the main source of drinking water for your household? (circle or tick on the options) | | | | | | | | | | | | | 1. Water from spring/ river/ pond 2. Dug well 3. Piped | | | | | | | | | | | | | | | | | | | |  | |
| 6 | What type of fuel does your household mainly use for Cooking?(code based on the respective number) | | | | | | | | | | | | | 1. Animal dung | | | | | | | | | | | | | | | | | | | |  | |
|  |  |  |  |  |  |  |  |  |  |  |  |  |  | 1. wood | | | | | | | | | | | | | | | | | | | |  | |
|  |  |  |  |  |  |  |  |  |  |  |  |  |  | 1. Charcoal | | | | | | | | | | | | | | | | | | | |  | |
|  |  |  |  |  |  |  |  |  |  |  |  |  |  | 1. Electricity | | | | | | | | | | | | | | | | | | | |  | |
| 7 | Does any member of this household have a bank or microfinance saving account (1=Yes, 0=N0) | | | | | | | | | | | | |  | | | | | | | | | | | | | | | | | | | |  | |
| 8 | How many of the following animals does the house hold have? (in number) | | | | | | | | | | | | | Milk Cows | | | | | | | | | | | | | | | | | | | |  | |
|  |  |  |  |  |  |  |  |  |  |  |  |  |  | Ox | | | | | | | | | | | | | | | | | | | |  | |
|  |  |  |  |  |  |  |  |  |  |  |  |  |  | Hen | | | | | | | | | | | | | | | | | | | |  | |
|  |  |  |  |  |  |  |  |  |  |  |  |  |  | Goat/Sheep | | | | | | | | | | | | | | | | | | | |  | |
|  |  |  |  |  |  |  |  |  |  |  |  |  |  | Donkey/Horse/Mule | | | | | | | | | | | | | | | | | | | |  | |
| 9 | Does this household own any agricultural land?  ( 1=Yes, 0=No) | | | | | | | | | | | | |  | | | | | | | | | | | | | | | | | | | |  | |
| 10 | How many hectares of agricultural land do members of this household own (in hectares) | | | | | | | | | | | | |  | | | | | | | | | | | | | | | | | | | |  | |
| 11 | Did you rent/lease out land over the last 12 months?  ( 1=Yes, 0=No) | | | | | | | | | | | | |  | | | | | | | | | | | | | | | | | | | |  | |
| 12 | In the past 12 months how many quintals did you got? [list amount produced for each crop] | | | | | | | | | | | | | 1. Teff | | | | | | | | | | | | | | | | | | | |  | |
|  |  |  |  |  |  |  |  |  |  |  |  |  |  | 2. Barely | | | | | | | | | | | | | | | | | | | |  | |
|  |  |  |  |  |  |  |  |  |  |  |  |  |  | 3. Wheat | | | | | | | | | | | | | | | | | | | |  | |
|  |  |  |  |  |  |  |  |  |  |  |  |  |  | 4. Maize | | | | | | | | | | | | | | | | | | | |  | |
| 13 | How much is monthly income of the family in birr? | | | | | | | | | | | | |  | | | | | | | | | | | | | | | | | | | |  | |
| **SECTION 2: MATERNAL (OBSTETRIC) AND NEONATAL CHARACTERSTICS** | | | | | | | | | | | | | | | | | | | | | | | | | | | | | | | | | | Code | |
| 201 | How many children do you have (parity)? | | | | | | | | ____________________ | | | | | | | | | | | | | | | | | | | | | | | | |  | |
| 202 | What was your last birth outcome during delivery | | | | | | | | 1. Still birth 2. Live birth 99. No response | | | | | | | | | | | | | | | | | | | | | | | | |  | |
| 203 | Did you have any history of neonatal death | | | | | | | | 1. Yes 2. No 99. No response | | | | | | | | | | | | | | | | | | | | | | | | |  | |
| 204 | What seems planning status of your last pregnancywhile you got pregnant for the last time? | | | | | | | | 1. I had a plan and desire to that pregnancy 2. The pregnancy occurred earlier than desired 3. The pregnancy occurred when no or more children were desired | | | | | | | | | | | | | | | | | | | | | | | | |  | |
| 205 | Did you have ANC visit while you were pregnant? | | | | | | | | 1. Yes 2. No  ______**if no go to 209** | | | | | | | | | | | | | | | | | | | | | | | | |  | |
| 206 | How many times you had got the visit? | | | | | | | |  | | | | | | | | | | | | | | | | | | | | | | | | |  | |
| 207 | Where was place of your last ANC visit? | | | | | | | | 1. Health center | | | | | | | | | | | 2. hospital | | | | | | | | | | | | | |  | |
|  |  |  |  |  |  |  |  |  | 3.health post | | | | | | | | | | | 4.other | | | | | | | | | | | | | |  |  |
| 208 | What about Course of your last pregnancy? | | | | | | | | 1. complicated 2. uncomplicated  99 No response ______**if 2 go to 211** | | | | | | | | | | | | | | | | | | | | | | | | |  | |
|  | If complicated ,What type of illness did you have sustained during last pregnancy | | | 1. Raise in blood pressure | | | | | | | | | | | | | | | 1. Gush of liquor | | | | | | | | | | | | | | |  | |
|  |  |  |  | 1. Vaginal Bleeding | | | | | | | | | | | | | | | 1. Anemia | | | | | | | | | | | | | | |  |  |
|  |  |  |  | 1. Persistent vomiting | | | | | | | | | | | | | | | 1. Severe headache | | | | | | | | | | | | | | |  |  |
|  |  |  |  | 1. Convulsion | | | | | | | | | | | | | | | 1. High grade fever | | | | | | | | | | | | | | |  |  |
| 209 | Where did you give your last birth? | | | 1. Health center | | | | | | | | | | | | | | | 1. Hospital | | | | | | | | | | | | | | |  | |
|  |  |  |  | 1. Health post | | | | | | | | | | | | | | | 1. Home | | | | | | | | | | | | | | |  |  |
|  |  |  |  | 1. Other specify | | | | | | | | | | | | | | |  | | | | | | | | | | | | | | |  |  |
| 210 | In what Mode of delivery you got your child? | | | 1.spontaneous vaginal delivery | | | | | | | | | | | | | | | | | | | | | | | | 2. Instrumental deliver | | | | | |  | |
|  |  |  |  | 3.Caesarean section | | | | | | | | | | | | | | | | | | | | | | | |  | | | | | |  |  |
| 211 | Had you faced any complication during the postpartum period? | | | | | | | | | | | | | | 1 Yes 2. No **if no skip to 212** | | | | | | | | | | | | | | | | | | |  | |
|  | What type of complication you faced? | | | 1. Abnormal vaginal bleeding | | | | | | | | | | | | | | | | | | 1. Convulsion | | | | | | | | | | | |  | |
|  |  |  |  | 1. Sepsis /high grade fever | | | | | | | | | | | | | | | | | | 1. Breast abnormalities | | | | | | | | | | | |  |  |
|  |  |  |  | 1. Swelling of the body (eye, face, extremities) | | | | | | | | | | | | | | | | | | 1. Severe abdominal pain | | | | | | | | | | | |  |  |
|  |  |  |  | 1. Others specify… | | | | | | | | | | | | | | | | | | 99 No response | | | | | | | | | | | |  |  |
| 212 | Had your baby faced any illness during postpartum period? | | | | | | | | | | | | | | | | | 1 Yes 2. No **if no skip to 212** | | | | | | | | | | | | | | | |  | |
|  | What type of complication  The newborn faced? | | | 1. Unable to breast feed | | | | | | | | | | | | | | | | | | | | | 1. Breathing difficulty | | | | | | | | |  | |
|  |  |  |  | 1. Sepsis | | | | | | | | | | | | | | | | | | | | | 1. hot/cold extremities | | | | | | | | |  |  |
|  |  |  |  | 1. Pus at umbilical area | | | | | | | | | | | | | | | | | | | | | 6. Vomiting | | | | | | | | |  |  |
|  |  |  |  | 1. Other | | | | | | | | | | | | | | | | | | | | |  | | | | | | | | |  |  |
| **SECTION THREE :- HEALTH SYSTEM RELATED FACTORS (ACCESS TO SKILLED MATERNITY CARE)** | | | | | | | | | | | | | | | | | | | | | | | | | | | | | | | | | | **Code** | |
| 301 | How long does it take you to walk to reach nearby health facility from your home? | | | | | | | | | | Minuit/hours [_______]  I don’t know---------------- | | | | | | | | | | | | | | | | | | | | | | |  | |
|  | What means of transport did you use while you were going to health facility for the last time?(More than one answer is possible) | | | | | | | | | | 1. on foot | | | | | | | | | | | | | | | | | | | 1. Vehicles | | | |  | |
|  |  |  |  |  |  |  |  |  |  |  | 1. Ambulance | | | | | | | | | | | | | | | | | | | 4. Strature | | | |  |  |
|  |  |  |  |  |  |  |  |  |  |  | 1. Others | | | | | | | | | | | | | | | | | | |  | | | |  |  |
| 302 | Who will decide when you want to go to health facility for maternity services? | | | | | | | | | | 1. Myself | | | | | | | | | | | | | | | | | | | 1. Me and my husband | | | |  | |
|  |  |  |  |  |  |  |  |  |  |  | 1. My husband | | | | | | | | | | | | | | | | | | |  | | | |  |  |
|  | Did you use maternity waiting room? | | | | | | | | | | 1. yes 2.no if yes skip to | | | | | | | | | | | | | | | | | | | | | | |  | |
|  | How long you stayed there? | | | | | | | | | | 1.for less than one week  2. one week 3.more than one week | | | | | | | | | | | | | | | | | | | | | | |  | |
| 303 | Are you member of women health development army (WHDA)? | | | | | | | | | | 1. Yes 2.No | | | | | | | | | | | | | | | | | | | | | | |  | |
| 304 | Are you model hose hold | | | | | | | | | | 1.yes (assure by observing certificate) 2.No | | | | | | | | | | | | | | | | | | | | | | |  | |
| **SECTION 4: KNOWLEDGE OF MOTHERS TOWARDS PNC SERVICE** | | | | | | | | | | | | | | | | | | | | | | | | | | | | | | | | | | | Code |
| 401 | Had you ever heard about post natal care service? | | | | | | | | | | | | | | | | 1. Yes 2. No | | | | | | | | | | | | | | | | | |  |
|  | From Where did you hear the information?  (More than one answer is possible) | | | | | 1. From the health professional | | | | | | | | | | | | | | | | | | | | | | | | | 1. From HEWs | | | |  |
|  |  |  |  |  |  | 1. From relatives | | | | | | | | | | | | | | | | | | | | | | | | | 1. From friends | | | |  |
|  |  |  |  |  |  | 1. From radio | | | | | | | | | | | | | | | | | | | | | | | | | 1. From TV | | | |  |
|  |  |  |  |  |  | 99.No response | | | | | | | | | | | | | | | | | | | | | | | | |  | | | |  |
| 402 | Do you know any advantage of PNC service? | | | | | | | | | | | 1. Yes 2. No 99. No response | | | | | | | | | | | | | | | | | | | | | | |  |
|  | What are the advantages of PNC service [at least one]?  (More than one answer is possible)  (Don’t mention choices) | | 1. To prevent any health problems of the mother related with delivery 2. To prevent any health problems of the baby after delivery 3. To get counseling on feeding practice of baby 4. To get counseling on importance of immunized baby 5. To get counseling on family planning | | | | | | | | | | | | | | | | | | | | | | | | | | | | | | | |  |
| 403 | Do you know the recommended visits for PNC? | | | | | | | | | | | | 1. Yes 2. No 99. No response | | | | | | | | | | | | | | | | | | | | | |  |
|  | How many times women should get PNC? | | | | | | | Number [________________________] | | | | | | | | | | | | | | | | | | | | | | | | | | |  |
| 404 | Do you know the correct timing of recommended PNC visits? | | | | | | | 1. Yes 2. No  99. No response | | | | | | | | | | | | | | | | | | | | | | | | | | |  |
|  | When the times of visits?(if she respond in the following manner, circle YES) | | | | 1. within the first 24 hr. -----for the first  2.On the third days to 6dys----for the second  3. on the 7-14 days------for the third visit  4. at 6^th^ week-------fourth visit | | | | | | | | | | | | | | | | | | | | | | | | | | | | | |  |
| 405 | Do you think PNC service is payable? | | | | | | | | | | | 1. Yes 2. No 99. No response | | | | | | | | | | | | | | | | | | | | | | |  |
| 406 | Do you know components of services given during post natal care visits? (If she can mention at least one component)? | | | | | | | | | | | | | | | | | | | | | | | 1. Yes 2. No  99. No response | | | | | | | | | | |  |
|  | **NB:** Yes= only if she can mention at least one content of care | | | | Body temperature measurement  Examination of abnormal bleeding  Counseling on Exclusive Breast feeding  Iron supplementation(IFA)  Blood pressure measurement  Cord care  Family planning services  Counseling about care of the baby….etc. | | | | | | | | | | | | | | | | | | | | | | | | | | | | | |  |
| 407 | Can you mention at least one maternal danger sign and symptoms during PPP? | | | | | | | | | | | | | | | | | | | | | | | 1. Yes 2. No  99. No response | | | | | | | | | | |  |
|  | **NB:** Yes=if she can mention at least one maternal danger sign | | | | | | | 1. Heavy bleeding 2. Abnormal body movement 3. high grade fever 4. Uterine tenderness 5. Lower abdominal pain 6. Foul vaginal discharge … | | | | | | | | | | | | | | | | | | | | | | | | | | |  |
| 408 | Do you know at least one new born danger sign and symptoms during PPP? | | | | | | | | | | | | | | | 1. Yes 2. No 99. No response | | | | | | | | | | | | | | | | | | |  |
|  | **NB:** Yes=if she can mention at least one danger signs in new born? | | | | 1. breast Feeding problem | | | | | | | | | | | | | | | | | | | | | | 2. fast breath/grunting | | | | | | | |  |
|  |  |  |  |  | 3. Abnormal body movement | | | | | | | | | | | | | | | | | | | | | | 4. Umbilical redness | | | | | | | |  |
|  |  |  |  |  | 5. Too cold or too hot | | | | | | | | | | | | | | | | | | | | | | Other… | | | | | | | |  |
| 409 | Do you know consequences of not attending adequate PNC visit? | | | | | | | | | 1. Yes 2. No 99. No response | | | | | | | | | | | | | | | | | | | | | | | | |  |
| 410 | From where you can got recommended PNC services? | | | | | | | | | 1. Yes 2. No 99. No response | | | | | | | | | | | | | | | | | | | | | | | | |  |
| 410 | Yes= if she know at least setup where PNC service is given | | | | | | | | | 1. At health center 2. At health post 3. At home level(within home visit) | | | | | | | | | | | | | | | | | | | | | | | | |  |
| **SECTION FIVE :- PRACTICE OF WOMEN ON CPNC SERVICES** | | | | | | | | | | | | | | | | | | | | | | | | | | | | | | | | | | | **Code** |
| 501 | After delivery of your last child did you ever get PNC service within The first six weeks? | | | | | | | | | | | | | | | | | | | | | | 1. Yes  2. no **if no skip to 503** | | | | | | | | | | | |  |
| 502 | How many times you got the service? | | | | | | _____________ | | | | | | | | | | | | | | | | | | | | | | | | | | | |  |
| 503 | Why you didn’t got the service  (More than one answer is possible)  (Don’t mention the choice) | | | | | | 1. Distance to the health facility is too long 2. Lack of transportation 3. Lack of awareness on its importance 4. Culture doesn’t allow to go away from home at this time 5. HWs didn’t come to home 6. Others specify**_____________________** | | | | | | | | | | | | | | | | | | | | | | | | | | | |  |
| 504 | What was your main reason while you were attending PNC?  (More than one answer is possible)  (Don’t mention the choices) | | | | | | 1. I feel sick after delivery | | | | | | | | | | | | | | | | | | | 1. My baby was sick | | | | | | | | |  |
|  |  |  |  |  |  |  | 1. Immunization of the baby | | | | | | | | | | | | | | | | | | | 1. To get family planning | | | | | | | | |  |
|  |  |  |  |  |  |  | 1. I know PNC follow up is important for me and my baby health | | | | | | | | | | | | | | | | | | | | | | | | | | | |  |
|  |  |  |  |  |  |  | 1. Other­­­­­­­__________ | | | | | | | | | | | | | | | | | | | | | | | | | | | |  |
| 505 | At what time were your visits?(fill the respected number and more than one answer is possible | | | | | | 1. within the first 24 hr. | | | | | | | | | | | | | | | | | | | | | | | | | | | |  |
|  |  |  |  |  |  |  | 1. on day 3- 6 | | | | | | | | | | | | | | | | | | | | | | | | | | | |  |
|  |  |  |  |  |  |  | 1. on 7-14 days | | | | | | | | | | | | | | | | | | | | | | | | | | | |  |
|  |  |  |  |  |  |  | 1. at 6^th^ week | | | | | | | | | | | | | | | | | | | | | | | | | | | |  |
|  |  |  |  |  |  |  |  | | | | | | | | | | | | | | | | | | | | | | | | | | | |  |
| 506 | By Whom your first PNC visit was delivered? | | | | | | 1. Health professionals | | | | | | | | | | | | | | | | | | | | | | 1. HEW | | | | | |  |
|  |  |  |  |  |  |  | 3. others | | | | | | | | | | | | | | | | | | | | | |  | | | | | |  |
|  | | | | | | | | | | | | | | | | | | | | | | | | | | | | | | | | | | | |
| 507 | Which service you got during service delivery (for those who got any PNC visit) | Response category | | | | | | | | | | | | | | | | | | | | | | | | | | | | | | 1=Yes | 2=No | |  |
|  |  | Body temperature measurement (maternal) | | | | | | | | | | | | | | | | | | | | | | | | | | | | | | 1 | 2 | |  |
|  |  | Breasts examination | | | | | | | | | | | | | | | | | | | | | | | | | | | | | | 1 | 2 | |  |
|  |  | Examination of abnormal bleeding | | | | | | | | | | | | | | | | | | | | | | | | | | | | | | 1 | 2 | |  |
|  |  | Counseling on Exclusive Breast feeding | | | | | | | | | | | | | | | | | | | | | | | | | | | | | | 1 | 2 | |  |
|  |  | Iron supplementation(IFA) | | | | | | | | | | | | | | | | | | | | | | | | | | | | | | 1 | 2 | |  |
|  |  | Blood pressure measurement | | | | | | | | | | | | | | | | | | | | | | | | | | | | | | 1 | 2 | |  |
|  |  | Family planning services | | | | | | | | | | | | | | | | | | | | | | | | | | | | | | 1 | 2 | |  |
|  |  | Counseling about HIV transmission | | | | | | | | | | | | | | | | | | | | | | | | | | | | | | 1 | 2 | |  |
|  |  | Counseling about care of the baby | | | | | | | | | | | | | | | | | | | | | | | | | | | | | | 1 | 2 | |  |
|  |  | Counseling on baby danger signs | | | | | | | | | | | | | | | | | | | | | | | | | | | | | | 1 | 2 | |  |
|  |  | Counseling on personal hygiene | | | | | | | | | | | | | | | | | | | | | | | | | | | | | | 1 | 2 | |  |
|  |  | Referral service | | | | | | | | | | | | | | | | | | | | | | | | | | | | | | 1 | 2 | |  |
|  |  | Immunization | | | | | | | | | | | | | | | | | | | | | | | | | | | | | | 1 | 2 | |  |
|  |  | cord care by using clorhexidine | | | | | | | | | | | | | | | | | | | | | | | | | | | | | | 1 | 2 | |  |
|  |  | Eye care | | | | | | | | | | | | | | | | | | | | | | | | | | | | | | 1 | 2 | |  |
|  |  | Body temperature measurement (NB) | | | | | | | | | | | | | | | | | | | | | | | | | | | | | | 1 | 2 | |  |
|  |  | Checking of the body weight | | | | | | | | | | | | | | | | | | | | | | | | | | | | | | 1 | 2 | |  |

**THANK YOU VERY MUCH!!!!!**

## Annex 5: Amharic Version of Interviewer Administered Questionnaire

የመጠይቁውጤት

| 1. የተማላ/ምሉዕ | | | | | | | | | | | | | | | | 1. በከፊልየተማላ | | | | | | | | | | | | | | | | | | | | | | | | | | |
| --- | --- | --- | --- | --- | --- | --- | --- | --- | --- | --- | --- | --- | --- | --- | --- | --- | --- | --- | --- | --- | --- | --- | --- | --- | --- | --- | --- | --- | --- | --- | --- | --- | --- | --- | --- | --- | --- | --- | --- | --- | --- | --- |
| 1. ያልተማላ | | | | | | | | | | | | | | | | 3. ሌላምክንያትካለይገለጽ | | | | | | | | | | | | | | | | | | | | | | | | | | |
| የተጠያቂውመለያቁጥር______ የመጠየቅመለያቁጥር______  ትዕዛዝ፡-ትክክለኛውንመልስአክብብ  **ክፍል1: የማህበራዊናሥነ- ህዝብመገለጫመጠይቅ** | | | | | | | | | | | | | | | | | | | | | | | | | | | | | | | | | | | | | | | | | | |
| ተ.ቁ | ጥያቄ | | | | | | | መልስ | | | | | | | | | | | | | | | | | | | | | | | | | | | | | | | | | ኮድ | |
| 101 | ዕድሜዎትስንትነው( በዓመት)? | | | | | | | 1. ----------------- 2. አላውቀውም 99. መልስየለም | | | | | | | | | | | | | | | | | | | | | | | | | | | | | | | | |  | |
| 102 | የጋብቻሁኔታ? | | | | | | | 1. ያገባች / በትዳርላይያለች | | | | | | | | | | | | | | | | | | | | | | | | | | | | 1. በፍቺየተለያዩ | | | | |  | |
|  |  |  |  |  |  |  |  | 1. ያላገባች | | | | | | | | | | | | | | | | | | | | | | | | | | | | 1. ባልየሞተባት | | | | |  |  |
|  |  |  |  |  |  |  |  | 99. መልስየለም | | | | | | | | | | | | | | | | | | | | | | | | | | | | | | | | |  |  |
| 103 | የየትኛውሀይማኖትተከታይኖት | | | | | | | 1. ኦርቶዶክስ | | | | | | | | | | | | | | | | | | | | | | | | | | | | 1. ሙስሊም | | | | |  | |
|  |  |  |  |  |  |  |  | 1. ፕሮቴስታንት | | | | | | | | | | | | | | | | | | | | | | | | | | | | 1. ካቶሊክ | | | | |  |  |
|  |  |  |  |  |  |  |  | 1. ሌላ | | | | | | | | | | | | | | | | | | | | | | | | | | | | 99 መልስየለም | | | | |  |  |
| 104 | ብሄሮትምንድነው | | | | | | | 1. ጉራጌ | | | | | | | | | | | | | | | | | | | | | | | | | | | | 1. አማራ | | | | |  | |
|  |  |  |  |  |  |  |  | 1. ኦሮሞ | | | | | | | | | | | | | | | | | | | | | | | | | | | | 99 መልስየለም | | | | |  |  |
| 105 | የትምህርትደረጃዎት? | | | | | | | 1. መደበኛትምህርትያላገኘች | | | | | | | | | | | | | | | | | | | | | | | | | 1. ከ1-8ኛ | | | | | | | |  | |
|  |  |  |  |  |  |  |  | 1. ከ9-12ኛ | | | | | | | | | | | | | | | | | | | | | | | | | 1. ኮሌጅናከዛበላይ | | | | | | | |  |  |
|  |  |  |  |  |  |  |  | 99 መልስየለም | | | | | | | | | | | | | | | | | | | | | | | | |  | | | | | | | |  |  |
| 106 | በአሁኑጊዜእርሶየሚሠሩትስራምንድንነው? | | | | | | | 1. የቤትእመቤት | | | | | | | | | | | | | | | | | | | | | | | | | 1. የመንግስትሰራተኛ | | | | | | | |  | |
|  |  |  |  |  |  |  |  | 1. ነጋዴ | | | | | | | | | | | | | | | | | | | | | | | | | 1. ቀንሰራተኛ | | | | | | | |  |  |
|  |  |  |  |  |  |  |  | 1. ግብርና/እርሻስራ | | | | | | | | | | | | | | | | | | | | | | | | | 99 መልስየለም | | | | | | | |  |  |
| 107 | የባልሽየትምህርትደረጃ? | | | | | | | 1. ኢ-መደበኛትምህርት | | | | | | | | | | | | | | | | | | | | | | | | 1. ከ1-8ኛ | | | | | | | | |  | |
|  |  |  |  |  |  |  |  | 1. ከ9-12ኛ | | | | | | | | | | | | | | | | | | | | | | | | 1. ኮሌጅናከዛበላይ | | | | | | | | |  |  |
|  |  |  |  |  |  |  |  | 99 መልስየለም | | | | | | | | | | | | | | | | | | | | | | | |  | | | | | | | | |  |  |
| 108 | በአሁኑጊዜባለቤትዎየሚሰሩትስራምንድነው? | | | | | | | 1. መንግስትስራ | | | | | | | | | | | | | | | | | | | | | | | | 1. የግልስራ | | | | | | | | |  | |
|  |  |  |  |  |  |  |  | 1. ነጋዴ | | | | | | | | | | | | | | | | | | | | | | | | 1. የቀንሰራተኛ | | | | | | | | |  |  |
|  |  |  |  |  |  |  |  | 1. .ግብርና/እርሻስራ | | | | | | | | | | | | | | | | | | | | | | | | 1. ስራየለውም | | | | | | | | |  |  |
| 109 | አጠቃላየቤተሰቡአባላትብዛትስንትነው? | | | | | | | | | | | | | | | |  | | | | | | | | | | | | | | | | | | | | | | | |  | |
| 110 | **የቤተሰቡየሃብትሁኔታአመላካችመጠይቅ** | | | | | | | | | | | | | | | | | | | | | | | | | | | | | | | | | | | | | | | | ኮድ | |
| 1 | ቤተሰቡእነዚህመገልገያቁሳቁሶች/ መሳሪዎችአሉት (አዎ=1 አይደለም=0) | | | | | | | | | | | ኤልክትሪክ | | | | | | | | | | | | | | | | | | | | | | | | | | | | |  | |
|  |  |  |  |  |  |  |  |  |  |  |  | ሬዲዮ | | | | | | | | | | | | | | | | | | | | | | | | | | | | |  | |
|  |  |  |  |  |  |  |  |  |  |  |  | ቴሌቪዘዥን | | | | | | | | | | | | | | | | | | | | | | | | | | | | |  | |
|  | በቁጥር | | | | | | | | | | | ጠረጴዛ | | | | | | | | | | | | | | | | | | | | | | | | | | | | |  | |
|  |  |  |  |  |  |  |  |  |  |  |  | ወንበር | | | | | | | | | | | | | | | | | | | | | | | | | | | | |  | |
|  |  |  |  |  |  |  |  |  |  |  |  | አልጋ | | | | | | | | | | | | | | | | | | | | | | | | | | | | |  | |
|  |  |  |  |  |  |  |  |  |  |  |  | ተንቀሳቃሽስልክ | | | | | | | | | | | | | | | | | | | | | | | | | | | | |  | |
|  |  |  |  |  |  |  |  |  |  |  |  | ሌላካለይገለጽ | | | | | | | | | | | | | | | | | | | | | | | | | | | | |  | |
| 2 | ከቤተሰቡአባለትቢያንስአንዱእንዚህንብረቶችአሉት | | | | | | | | | | | ሳይክል | | | | | | | | | | | | | | | | | | | | | | | | | | | | |  | |
|  |  |  |  |  |  |  |  |  |  |  |  | ሞተር | | | | | | | | | | | | | | | | | | | | | | | | | | | | |  | |
|  |  |  |  |  |  |  |  |  |  |  |  | ባጃጅ | | | | | | | | | | | | | | | | | | | | | | | | | | | | |  | |
|  |  |  |  |  |  |  |  |  |  |  |  | መኪና | | | | | | | | | | | | | | | | | | | | | | | | | | | | |  | |
| 3 | ቤተሰቡየግልመኖሪያቤትአለው | | | | | | | | | | | 1. አዎ 2. አይደለም 99 መልስየለም | | | | | | | | | | | | | | | | | | | | | | | | | | | | |  | |
| 4 | የቤቱጣራየተሰራውከምንድነው(በማየት) | | | | | | | | | | | 1.ከሳር | | | | | | | | | | | | | | | | | | | | | | | | | | | | |  | |
|  |  |  |  |  |  |  |  |  |  |  |  | 2. ከቆርቆሮ | | | | | | | | | | | | | | | | | | | | | | | | | | | | |  | |
| 5 | የምትጠቀሙትውሃከየትነውምታገኙት(ቁጥሩንያስቀምጡ) | | | | | | | | | | | 1.ከወንዝዝ/ከኩሬ | | | | | | | | | | | | | | | | | | | | | | | | | | | | |  | |
|  |  |  |  |  |  |  |  |  |  |  |  | 2. የጉድጋድውሃ | | | | | | | | | | | | | | | | | | | | | | | | | | | | |  | |
|  |  |  |  |  |  |  |  |  |  |  |  | 3. የባንባውሃ | | | | | | | | | | | | | | | | | | | | | | | | | | | | |  | |
| 6 | ምግብለማብሰልየምትጠቀሙት(ቁጥሩንያስቀምጡ)? | | | | | | | | | | | 1. ኩበት | | | | | | | | | | | | | | | | | | | | | | | | | | | | |  | |
|  |  |  |  |  |  |  |  |  |  |  |  | 1. እንጨት | | | | | | | | | | | | | | | | | | | | | | | | | | | | |  | |
|  |  |  |  |  |  |  |  |  |  |  |  | 1. ከሰል | | | | | | | | | | | | | | | | | | | | | | | | | | | | |  | |
|  |  |  |  |  |  |  |  |  |  |  |  | 1. ኤሌክትሪክ | | | | | | | | | | | | | | | | | | | | | | | | | | | | |  | |
| 7 | ከቤተሰቡአባለትውስጥየባንክ/የኦሞማይክሮፋይናንስቁጠባደብተርያለውአለ(አዎ=1 አይደለም=0) | | | | | | | | | | |  | | | | | | | | | | | | | | | | | | | | | | | | | | | | |  | |
| 8 | ቤተሰቡምናክልየቤትእንስሳቶችአሉት?(በቁጥር) | | | | | | | | | | | የወተወትላም | | | | | | | | | | | | | | | | | | | | | | | | | | | | |  | |
|  |  |  |  |  |  |  |  |  |  |  |  | በሬ | | | | | | | | | | | | | | | | | | | | | | | | | | | | |  | |
|  |  |  |  |  |  |  |  |  |  |  |  | ዶሮ | | | | | | | | | | | | | | | | | | | | | | | | | | | | |  | |
|  |  |  |  |  |  |  |  |  |  |  |  | በግ/ፍየል | | | | | | | | | | | | | | | | | | | | | | | | | | | | |  | |
|  |  |  |  |  |  |  |  |  |  |  |  | ፈረስ/በቅሎ/አህያ | | | | | | | | | | | | | | | | | | | | | | | | | | | | |  | |
| **በባለፉት 12 ወራትየነበረውየመሬትአጠቃቀምናየሰብልምርትሁኔታ.** | | | | | | | | | | | | | | | | | | | | | | | | | | | | | | | | | | | | | | | | |  | |
| 9 | ቤተሰቡየእርሻመሬትአለው?(አዎ=1 አይደለም=0) | | | | | | | | | | | | | | | | | | | | | | | | | | | | | | | | | | | | | | | |  | |
| 10 | ለእርሻአገልግሎትየሚውልመሬትበሄክታር? | | | | | | | | | | | | | | | | | 1. --------------- 2. አይታወቅም | | | | | | | | | | | | | | | |  | | | | | | |  | |
| 11 | በዚህአመትውስትለእርሻየሚውልመሬትአከራይታቹሃል? (አዎ=1 አይደለም=0) | | | | | | | | | | | | | | | | |  | | | | | | | | | | | | | | | | | | | | | | |  | |
| 12 | ከባለፈውአመትጀምሮበዋናነትካመረታቹትምርትየተገኘውምርትበኩንታል? | | | | | | | | | | | | | | | | | 1. ጤፍ | | | | | | | | | | | | | | | | | | | | | | |  | |
|  |  |  |  |  |  |  |  |  |  |  |  |  |  |  |  |  |  | 1. ገብስ | | | | | | | | | | | | | | | | | | | | | | |  | |
|  |  |  |  |  |  |  |  |  |  |  |  |  |  |  |  |  |  | 1. ስንዴ | | | | | | | | | | | | | | | | | | | | | | |  | |
|  |  |  |  |  |  |  |  |  |  |  |  |  |  |  |  |  |  | 1. በቆሎ | | | | | | | | | | | | | | | | | | | | | | |  | |
|  |  |  |  |  |  |  |  |  |  |  |  |  |  |  |  |  |  | 1. ቡና | | | | | | | | | | | | | | | | | | | | | | |  | |
| 13 | አማካኝየቤተሰቡወርሃዊገቢስንትነው?(በብር | | | | | | | | | | | | | | | | | 1. 2 አላውቅም | | | | | | | | | | | | | | | | | | | | | | |  | |
| **ክፍል 2:የዕናትዬዋናየህጻኑ (በእርግዝና፣በወሊድናበድህረወሊድወቅት )የነበሩየጤናሁነቶች** | | | | | | | | | | | | | | | | | | | | | | | | | | | | | | | | | | | | | | | | |  | |
| 201 | ስንትልጆችአሉሽ? | | | | | | | | | | | | | | | | | | | | ____________________ | | | | | | | | | | | | | | | | | | | |  | |
| 202 | ለመጨረሻጊዜስትወልጂየወሊድዉጤትምንነበር? | | | | | | | | | | | | | | | | | | | | 1.በሂወትየተወለደ 2. ሞቶየተወለደ 99 መልስየለም | | | | | | | | | | | | | | | | | | | |  | |
| 203 | ከዚህበፊትበወለድሽበመጀመሪያውወርውስጥልጅሞቶብሽያውቃል? | | | | | | | | | | | | | | | | | | | | 1. አዎ 2. አይደለም | | | | | | | | | | | | | | | | | | | |  | |
| 204 | ለመጨረሻጊዜስታረግዢየነበረሽየእርግዝና እቅድ ሁኔታ? | | | | | | | | | | | | | | | | | | | | 1. ለመውለድ ፍላጎትና እቅድ ነበረኝ 2. መውለድ ከምፈልግበት ጊዜ ቀድሜ ነበር ያረገዝኩት 3. በዛ ወቅት ምንም አይነት ወይም ተጨማሪ ልጅ እንዲኖረኝ አልፈልግም ነበር | | | | | | | | | | | | | | | | | | | |  | |
| 205 | ቅድመወሊድክትትልአርገሽነበር? | | | | | | | | | | | | | | | | | | | | 1. አዎ 2. አይደለም **______**አይደለምከሆነወደቁጥር 208 | | | | | | | | | | | | | | | | | | | |  | |
| 206 | ለምንያክልጊዜክትትልአድረገሻል? | | | | | | | | | | | | | | | | | | | |  | | | | | | | | | | | | | | | | | | | |  | |
| 207 | የትነበርለመጨረሻጊዜቅድመወሊድክትትልያረግሺው? | | | | | | | | | | | | | | | | | | | | 1. ጤናጣቢያ | | | | | | | | | | | | | | 2. ሆስፒታል | | | | | |  | |
|  |  |  |  |  |  |  |  |  |  |  |  |  |  |  |  |  |  |  |  |  | 3. ጤናኬላ | | | | | | | | | | | | | | 4. ሌላቦታ | | | | | |  | |
|  |  |  |  |  |  |  |  |  |  |  |  |  |  |  |  |  |  |  |  |  | 99መልስየለም | | | | | | | | | | | | | |  | | | | | |  | |
| 208 | በመጨረሻውእርግዝናሽወቅትየነበረውየጤናሽሁኔታምንይመስልነበር? | | | | | | | | | | | | | | | | | | | | 1.የተወሳሰበ 2.ያልተወሳሰበ  መልሱ 2 ከሆነወደቁጥር 210 | | | | | | | | | | | | | | | | | | | |  | |
| 209 | በእርግዝናሽወቅትምንአይነትየጤናእክል/መወሳሰብገጥሞሽነበር(በጤናባለሙያየተነገራትምካለይጠቀስ)? | | | 1. የደምግፊትመጨመር | | | | | | | | | | | | | | | | | | | | | 1. የእንሽርትውሃቀድሞመፍሰስ | | | | | | | | | | | | | | | |  | |
|  |  |  |  | 1. የደምመፍሰስ | | | | | | | | | | | | | | | | | | | | | 1. የደምማነስበሽታ | | | | | | | | | | | | | | | |  |  |
|  |  |  |  | 1. ከፍተኛትውከት | | | | | | | | | | | | | | | | | | | | | 1. ማንቀጥቀት/ራስንመሳት | | | | | | | | | | | | | | | |  |  |
|  |  |  |  | 1. ከፍተኛትኩሳት | | | | | | | | | | | | | | | | | | | | | 1. ከፍተኛራስምታት 2. … ሌላምካለይጠቀስ | | | | | | | | | | | | | | | |  |  |
| 210 | የመጨረሻውልጅሽየተገላገልሽውየትነበር? | | | 1. በጤናጣቢያ | | | | | | | | | | | | | | | | | | | | | | | | | 1. በሆስፒታል | | | | | | | | | | | |  | |
|  |  |  |  | 1. በጤናኬላ | | | | | | | | | | | | | | | | | | | | | | | | | 1. እቤት | | | | | | | | | | | |  | |
|  |  |  |  | 1. በግልከሊኒክ | | | | | | | | | | | | | | | | | | | | | | | | | ሌላ……. | | | | | | | | | | | |  | |
| 211 | በምንአይነትመንገድነውልጅሽንየወለድሽው? | | | 1. በማህጸንበር | | | | | | | | | | | | | | | | | | | | | | | | | 1. በመሳሪያታግዤ/ሰቲች | | | | | | | | | | | |  | |
|  |  |  |  | 3. በቀዶህክምና(በኦፕራሲዮን) | | | | | | | | | | | | | | | | | | | | | | | | | | | | | | | | | | | | |  | |
| 212 | ከወሊድበሓላባሉጊዜያትየተወሳሰበየጤናችግር/እክልገጥሞሽነበር ? | | | | | | | | | | | | 1 አዎ 2. አይደለም  _____አይደለምከሆነውደቁጥር 214 | | | | | | | | | | | | | | | | | | | | | | | | | | | |  | |
| 213 | ምንአይነትየጤናችግርገጥሞሽነበር? | | | | | 1. ከወሊድበኃላከፍተናየደምመፍሰስ | | | | | | | | | | | | | | | | | | | | | | 1. ማንቀጥቀጥናራስንመሳት | | | | | | | | | | | | |  | |
|  |  |  |  |  |  | 1. ከፍተኛትኩሳት | | | | | | | | | | | | | | | | | | | | | | 1. ከጡትጋተያያዥህመሞች | | | | | | | | | | | | |  | |
|  |  |  |  |  |  | 1. የሰውነትእብጠት | | | | | | | | | | | | | | | | | | | | | | ከፍተኛየሆድቁርጠት | | | | | | | | | | | | |  | |
|  |  |  |  |  |  | 1. ሌላካለይገለጽ… | | | | | | | | | | | | | | | | | | | | | |  | | | | | | | | | | | | |  |  |
| 214 | ከወሊድበሃላበነበሩቀናትልጅሽየተወሳሰበየጤናችግር/እክልገጥሞት/ገጥማትነበር ? | | | | | | | | | | | | | | 1 አዎ 2. አይደለም | | | | | | | | | | | | | | | | | | | | | | | | | |  | |
| 215 | ምንአይነትየጤናእክል? | | | | | | | | | | | | | | 1. ጡትያለመጥባትችግር | | | | | | | | | | | | | | | | | | | | | | | 1. የአተነፋፈስችግር | | |  | |
|  |  |  |  |  |  |  |  |  |  |  |  |  |  |  | 1. .ከፍተኛትኩሳት | | | | | | | | | | | | | | | | | | | | | | | 1. .ትውከት | | |  |  |
|  |  |  |  |  |  |  |  |  |  |  |  |  |  |  | 1. ሌላካለይገለጽ… | | | | | | | | | | | | | | | | | | | | | | |  | | |  |  |
| **ክፍልሶስት፡-ከጤናውስርዓትጋርተያያዥጉዳዮች (በሰለጠነባለሙያለሚሰጡየእናቶችጤናአገልግሎትተደራሽነት)** | | | | | | | | | | | | | | | | | | | | | | | | | | | | | | | | | | | | | | | | | | |
| 301 | የጤናአገልገሎትለማግኘትወደጤናተቃምለመድረስምንያክልጊዜይፈጅብሻል? | | | | | | | | | | | | | | | | | | | | | | በደቂቃ/በሰዓት [_______] | | | | | | | | | | | | | | | | | | | |
|  | የምትጠቀሚውየመጋጋዣአይነት?  (ከአንድበላመልስይቻላል) | | | | | | | | 1. በእግር | | | | | | | | | | | | | | 1. የህዝብ(መኪና/በባጃጅ/ሞተርሳክይል) | | | | | | | | | | | | | | | | | | |  |
|  |  |  |  |  |  |  |  |  | 1. አምቡላንስ | | | | | | | | | | | | | | 1. የግልተሸከርካሪ | | | | | | | | | | | | | | | | | | |  |
|  |  |  |  |  |  |  |  |  | 1. ቃሬዛ | | | | | | | | | | | | | | 6. ሌላካለይገለጽ………………… | | | | | | | | | | | | | | | | | | |  |
| 302 | ወደጤናተቃምለመሄድስትፈልጊየሚወስነውማነው? | | | | | | | | | 1. በራሴውሳኔ | | | | | | | | | | | | | | | | | | | | 1. በኔናበባለቤቴየጋራስምምነት | | | | | | | | | | | |  |
|  |  |  |  |  |  |  |  |  |  | 1. በባለቤቴውሳኔ | | | | | | | | | | | | | | | | | | | | 1. በቤተሰቦቼ | | | | | | | | | | | |  |
|  |  |  |  |  |  |  |  |  |  | 1. ሌለካለይገለጽ | | | | | | | | | | | | | | | | | | | |  | | | | | | | | | | | |  |
| 303 | በእረግዝናሽወቅትየእረጉዝእናቶችማረፍያክፍልቆይተሸነበር? | | | | | | | | | 1. አዎ 2.አይደለም-------አይደለምከሆነወደ 304 | | | | | | | | | | | | | | | | | | | | | | | | | | | | | | | |  |
|  | ለምንአክልጊዜቆየሽ? | | | | | | | | | 1. 1.ከአንድሳምነትበታች | | | | | | | | | | | | | | | | | | | | | | | | | | | 1. ለአንድሳምነት | | | | |  |
|  |  |  |  |  |  |  |  |  |  | 1. ከአንድሳምነትበላይ | | | | | | | | | | | | | | | | | | | | | | | | | | |  | | | | |  |
| 304 | የሴቶችጤናልማትሰራዊታአባልነሽ? | | | | | | | | | 1. አዎ 2.አይደለሁም | | | | | | | | | | | | | | | | | | | | | | | | | | | | | | | |  |
| 305 | ሞዴልእማወራነሽ? | | | | | | | | | 1.አዎ (የሞዴልቤተሰብነትመረጋገጫሰርትፊኬትበማየት) 2.አይደለሁም | | | | | | | | | | | | | | | | | | | | | | | | | | | | | | | |  |
| **ክፍልአራት: እናትዬዋበድህረወሊድአገልግሎትዙሪያያላትግንዛቤ** | | | | | | | | | | | | | | | | | | | | | | | | | | | | | | | | | | | | | | | | | | |
| 401 | ስለድህረወሊድአገልግሎትሰምተሽታውቂያለሽ? | | | | | | | | | 1. አዎ 2. አይደለምአይደለምከሆነወደ 402 | | | | | | | | | | | | | | | | | | | | | | | | | | | | | | | |  |
|  | ስለአገልግሎቱየሰሙትከማንነው?  (ከአንድበላይምላሽይቻላል) | | | | | | | | | 1. ከጤናባለሙያዎች | | | | | | | | | | | | | | | | | | | | | | | | | | | | | 1. ከጋደኞቼ | | |  |
|  |  |  |  |  |  |  |  |  |  | 1. ከዘመድ | | | | | | | | | | | | | | | | | | | | | | | | | | | | | 1. ከጎረቤት | | |  |
|  |  |  |  |  |  |  |  |  |  | 1. ከጤናኤክስቴንሽንባለሙያዎች | | | | | | | | | | | | | | | | | | | | | | | | | | | | | 1. በሬድዮ | | |  |
|  |  |  |  |  |  |  |  |  |  | 1. በቴሌቪዥን | | | | | | | | | | | | | | | | | | | | | | | | | | | | | 8. ሌላካለይገለጽ____ | | |  |
| 402 | የድህረወሊድአገልግሎትጥቅምታወቂያለሽ? | | | | | | | | | | | | | | | | | | | 1. አዎ 2. አላውቅም-99. መልስየለም | | | | | | | | | | | | | | | | | | | | | |  |
|  | የድህረ-ወሊድከትትልማድረግጥቅሞችምንድናቸው ? (ከአንድበላይምላሽይቻላል)  (ምረጫዎቹአይንገሩ) | 1.በእናትዬዋከወሊድጋርተያይዘውሊከሰቱየሚችሉየጤናእክሎችን  ለመለየትናለመከላከል  2.በህጻኑላይየሚከሰቱየጤናችግሮችንለመከላከል  3. በህጸኑአመጋገብዙሪያየምክርአገልግሎትለማግኘት  4. ህጸኑተገቢውንየክትባትአገልግሎትእንዲያገኝይረዳል  5. በቤተሰብምጣኔአገልግሎትዙሪያየምክርገልገሎትለማግኘትይረዳል  6. በግልንጽህናአጠባበቅዙሪያበቂግንዛቤእነዲኖርያስችላል  7. ሌላካለይገለጽ | | | | | | | | | | | | | | | | | | | | | | | | | | | | | | | | | | | | | | | |  |
|  | ከነዚህውስጥቢያንስአንዱጥቅምካወቀች (አዎየሚለውላይያክብቡ) | | | | | | | | | | | | | | | | | | | | | | | | | | | | | | | | | | | | | | | | |  |
| 403 | የተማላየየድህረ-ወሊድአገልግሎትለማግኘትስንትጉብኝትእንደሚያስፈልግታውቂያለሽ? | | | | | | | | | | | | | | | | | | 1. አዎ 2. አይደለም_______ከሆነወደ 404 | | | | | | | | | | | | | | | | | | | | | | |  |
|  | አንድእናትከወለደችበኃላለምንያክልጊዜየድህረወሊድክትትል/ጉብንትማግኘትአለባት? | | | | | | | | | | | | | | | | | | በቁጥር[________________________]  99 መልስየለም | | | | | | | | | | | | | | | | | | | | | | |  |
|  | ሶስትናከዛበላይየሚልውንከመለሰች (አዎየሚለውንያክብቡ) | | | | | | | | | | | | | | | | | | | | | | | | | | | | | | | | | | | | | | | | |  |
| 404 | ትክከለኛውየክትትልጊዜያቶችታውቂያለሽ | | | | | | | | | | | | | | | | | | 1. አዎ 2. አይደለም 99 መልስየለም | | | | | | | | | | | | | | | | | | | | | | |  |
|  | የጉብኝት/የክትትልየጊዜሰሌዳመችመችናቸው? | | 1. በ48 ሰዓታትውስትለመጀመሪያውክትትል | | | | | | | | | | | | | | | | | | | | | | | | 1. ከ3-7ኛቀንለሁለተኛውክትትል | | | | | | | | | | | | | | |  |
|  |  |  | 1. ከ7-14ኛውቀንለሶስተኛውክትትል | | | | | | | | | | | | | | | | | | | | | | | | 1. 6ኛውሳምንትለአራተኛውክትትል | | | | | | | | | | | | | | |  |
|  | ከላይበተጠቀሰውመሰረትከመለሰችአዎየሚለውንያክብቡ | | | | | | | | | | | | | | | | | | | | | | | | | | | | | | | | | | | | | | | | | |
| 405 | የድህረወሊድአገልግሎትለማግኘትያስከፍላል? | | | | | | | | | | | | | | | | | | | | | | | 1. አዎ 2. አላቅም 99 መልስየለም | | | | | | | | | | | | | | | | | |  |
| 406 | በድህረወሊድጉብኝትወቅትየሚሰጡአገልግሎቶችታወቂያለሽ? | | | | | | | | | | | | | | | | | | | | | | | 1. አዎ 2. አላቅም 99 መልስየለም | | | | | | | | | | | | | | | | | |  |
|  | ከሚሰጡአገልግሎቶችቢያንስአንዱንመጥቀስከቻለችብቻአዎሚለውንያክብቡ | | 1. ከልጁአመጋገብጋርበተያያዘግንዛቤይሰጣል 2. የደምማነስመድሃኒትይሰጣል 3. የቤተሰብምጣኔአገልግሎት 4. በልጅእንክብካቤዙሪያየምክርአገልግሎት 5. በህጻኑላይስለሚኖሩአደገኛምልክቶችየምክርአገልግሎት 6. በግልንጽህናአጠባበቅዙሪያየምክርአገልግሎት 7. ህጻኑከታመመህክምናይሰጣል 8. ወደሌላጤናተቃምእንድሄድተደርጋል(ሪፈርተደርጌ) 9. የክትባትአገልግሎት 10. ሰውነትክብደትልኬትይደረጋል 11. ሌላምካለይጠቀስ…… | | | | | | | | | | | | | | | | | | | | | | | | | | | | | | | | | | | | | | |  |
| 407 | ከወሊድበኃላበእናትዬዋላይስለሚከሰቱአደገኛምልክቶችታወቂለሽ? | | | | | | | | | | | | | | | | | | | | | | | | | 1. አዎ 2. አላቅም 99 መልስየለም | | | | | | | | | | | | | | | |  |
|  | ከነዚህምልክቶችቢያንስአንዱንመጥቀስከቻለችብቻአዎሚለውንያክብቡ | | | | | | 1. ከፍተኛየደምመፍሰስ | | | | | | | | | | | | | | | | | | | | | | | | እራስንመሳት | | | | | | | | | | |  |
|  |  |  |  |  |  |  | 1. ማንቀጥቀጥ | | | | | | | | | | | | | | | | | | | | | | | | ከፍተኛየሆነየሰውነትሙቀት | | | | | | | | | | |  |
|  |  |  |  |  |  |  | 1. ከፍተኛየሆድቁርጠት | | | | | | | | | | | | | | | | | | | | | | | | መጥፎጠረንያለውየማህጸንፈሳሸ | | | | | | | | | | |  |
| 408 | ከወሊድበኃላበህጻኑላይስለሚከሰቱአደገኛምልክቶችታወቂለሽ? | | | | | | | | | | | | | | | | | | | | | | 1.አዎ 2. አላውቅም  99 መልስየለም | | | | | | | | | | | | | | | | | | |  |
|  | ከነዚህምልክቶችቢያንስአንዱንመጥቀስከቻለችብቻአዎሚለውንያክብቡ | | | | | | | | | | 1. የአመጋገብችግር/ጡትአላመጥባት | | | | | | | | | | | | | | | | | | | | | | | | | | | | | | |  |
|  |  |  |  |  |  |  |  |  |  |  | 1. አላስፈላጊየአካልእንቅስቃሴ/ማንቀጥቀጥ | | | | | | | | | | | | | | | | | | | | | | | | | | | | | | |  |
|  |  |  |  |  |  |  |  |  |  |  | 1. ከፍተኛሙቀት/ብርድ | | | | | | | | | | | | | | | | | | | | | | | | | | | | | | |  |
|  |  |  |  |  |  |  |  |  |  |  | 1. ፈጠንአተነፋፈስ/ማቃሰት | | | | | | | | | | | | | | | | | | | | | | | | | | | | | | |  |
|  |  |  |  |  |  |  |  |  |  |  | 1. የዕምብርትአካባቢመቅላት | | | | | | | | | | | | | | | | | | | | | | | | | | | | | | |  |
|  |  |  |  |  |  |  |  |  |  |  | 1. ሌላካለይገለጽ__________________ | | | | | | | | | | | | | | | | | | | | | | | | | | | | | | |  |
| 409 | የተማላ/በቂየድህረወሊድአገልግሎትአለማግኘትሚያመጠቸውችግሮችታወቂያለሽ | | | | | | | | | | | | | | | | | | | | | 1.አዎ 2. አላውቅም 99 መልስየለም | | | | | | | | | | | | | | | | | | | |  |
|  | አዎከሆነአገልግሎቱካለማግኘትየሚመጡችግሮችምንምንናቸው(ቢያንስአንዱ) | | | | | | | | | | 1. በእናትዬዋህምምናሞትሊስከትልይችላል 2. በህጻኑላይህመምናሞትሊስከትልይችላል 3. ላላስፈላጊየህክምናወጪዎችሊደርግይችላል | | | | | | | | | | | | | | | | | | | | | | | | | | | | | | |  |
| 410 | ተገቢውንየድህረወሊድአገልግሎትከየትማግኘትተችያለሽ | | | | | | | | | | 1. ከጤናጣቢያ | | | | | | | | | | | | | | | | | | | | | | | | | | | | | 1. ከጤናኬላ | |  |
|  |  |  |  |  |  |  |  |  |  |  | 1. በቤትለቤትጉብኝት | | | | | | | | | | | | | | | | | | | | | | | | | | | | | 1. ሆስፒታል | |  |
|  |  |  |  |  |  |  |  |  |  |  | 99. መልስየለም | | | | | | | | | | | | | | | | | | | | | | | | | | | | |  | |  |
| **ክፍልአምስት: ከድህረ-ወሊድአገልግሎትተጠቃሚነትጋርየተያያዙሁነቶች** | | | | | | | | | | | | | | | | | | | | | | | | | | | | | | | | | | | | | | | | | |  |
| 501 | የመጨረሻውልጅሽከወለድሸበኃላሙሉየድህረ-ወሊድአገልግሎትአግኝተሽነበር ? | | | | | | | | | | | | | 1. አዎ  2. አላገኘሁም ------ከሆነወደቁጥር 503 | | | | | | | | | | | | | | | | | | | | | | | | | | | |  |
|  | ለስንትጊዜ/ዙርአገኘሽ? | | | | | | | | | | | | |  | | | | | | | | | | | | | | | | | | | | | | | | | | | |  |
|  | ሶስትናከዛበላይጉብኝትካገኘችአዎየሚለውንያክብቡ | | | | | | | | | | | | | | | | | | | | | | | | | | | | | | | | | | | | | | | | | |
| 502 | የተማላየድህረ-ወሊድአገልግሎትያላገኘሺበትምክንያትምንድነው  (ከአንድበላምላሽይቻላል)  (ምርጫውአይነገርም) | | | | | | | | | | | | | 1. ከጤናተቃሙያለውመንገድስለሚርቅ 2. የትራንስፖርትችግር 3. የግንዛቤእጥረት 4. የባህሉተጽእኖበዛወቅትከቤትእንድወጣአይፈቅድም 5. ጤናኤክስቴንሽንባለሙያዎችበቅርበትአላገኘሁም 6. ሌላካለይጠቀስ | | | | | | | | | | | | | | | | | | | | | | | | | | | |  |
| 504 | የድህረወሊድክትትልእነድታገኚምክንያትሽምንነበር(ከአንድበላይምላሽይቻላል)  (ምርጫውአይነገርም) | | | | | | | | | | | | | 1. ከወሊድበሃላየህምመስሜትስለነበረኝ  2. ህጻኑታሞስለነበር  3. ህጻኑንለማስከተብ 4. የቤተሰብምታኔአገልግሎትለማግኘት  5. የድህረ-ወሊድአገልግሎትማግኘቱለኔናለልጄጥቅምእንዳለውስለማወቅ 6. ጤናኤክስቴነሽንሰራተኞችወደቤትመጥተውአገልግሎቱስለሰጡኝ 7. ሌላካለይገለጽ | | | | | | | | | | | | | | | | | | | | | | | | | | | |  |
| 505 | አከልግሎቱያገኘሽባቸውጊዜያት? | | | | | | | | | | | | | 1.በመጀመሪያው24ሰዓታት(ለመጀመሪያውጉበረኝት/ክትትል  2.ከ 3-6ኛውቀን(ለሁለተኛውጉብኝት/ክትትል)  3. ከ7-14ኛውቀን (ለሶስተኛውጉብኝት/ክትትል  4. በ6ኛውሳምንት (ለአራተኛውጉብኝት/ክትትል) | | | | | | | | | | | | | | | | | | | | | | | | | | | |  |
| 506 | አገልግሎቱያገኘሽውበማንነው? | | | | | | | | | | | | | 1. በዶክተር 2.በአዋላጅነርሶች  3. በነርሶች 4. በጤናኤክስቴነሽንሰራተኞች 5. ሌላካለገለጽ | | | | | | | | | | | | | | | | | | | | | | | | | | | |  |
| 507 | በጉብኝት/በክትትልወቅትያገኘሻቸውአገልገሎቶችምንምንናቸው?  (ከአንድበላይምላሽይቻላል) | | | | የሰውነትሙቀትመለካት | | | | | | | | | | | | | | | | | | | | | | | | | | | | | | | | | | | | |  |
|  |  |  |  |  | የጡትምርመራ | | | | | | | | | | | | | | | | | | | | | | | | | | | | | | | | | | | | |  |
|  |  |  |  |  | የደምመፍሰስመኖርአለመኖሩመረጋገጥ | | | | | | | | | | | | | | | | | | | | | | | | | | | | | | | | | | | | |  |
|  |  |  |  |  | ከጡትዉጪሌላምግብማስጀመርእንደሌለብንምክርአግኝቻለው | | | | | | | | | | | | | | | | | | | | | | | | | | | | | | | | | | | | |  |
|  |  |  |  |  | የደምማነስመከላከያመድሀኒትተሰጥቶኛል | | | | | | | | | | | | | | | | | | | | | | | | | | | | | | | | | | | | |  |
|  |  |  |  |  | የደምግፊትልኬትአግኝችላው | | | | | | | | | | | | | | | | | | | | | | | | | | | | | | | | | | | | |  |
|  |  |  |  |  | የቤተሰብምጣኔአገልግሎት | | | | | | | | | | | | | | | | | | | | | | | | | | | | | | | | | | | | |  |
|  |  |  |  |  | በህጻኑላይስለሚኖሩአደገኛምልክቶችየምክርአገልግሎት | | | | | | | | | | | | | | | | | | | | | | | | | | | | | | | | | | | | |  |
|  |  |  |  |  | በግልንጽህናአጠባበቅዙሪያየምክርአገልግሎት | | | | | | | | | | | | | | | | | | | | | | | | | | | | | | | | | | | | |  |
|  |  |  |  |  | ወደሌላጤናተቃምእንድሄድተደርጋል(ሪፈርተደርጌ) | | | | | | | | | | | | | | | | | | | | | | | | | | | | | | | | | | | | |  |
|  |  |  |  |  | የክትባትአገልግሎት | | | | | | | | | | | | | | | | | | | | | | | | | | | | | | | | | | | | |  |
|  |  |  |  |  | የቆዳናየእብርትአካባቢንጽህናኤዝሁኔታንማረጋገጥ | | | | | | | | | | | | | | | | | | | | | | | | | | | | | | | | | | | | |  |
|  |  |  |  |  | የእትብ/እምብርትአካባቢየምስራችተቀብታል | | | | | | | | | | | | | | | | | | | | | | | | | | | | | | | | | | | | |  |
|  |  |  |  |  | የአይንንጽህናአያያዝ | | | | | | | | | | | | | | | | | | | | | | | | | | | | | | | | | | | | |  |
|  |  |  |  |  | ሰውነትሙቀትልኬት | | | | | | | | | | | | | | | | | | | | | | | | | | | | | | | | | | | | |  |
|  |  |  |  |  | ሰውነትክብደትልኬትተድረጋል | | | | | | | | | | | | | | | | | | | | | | | | | | | | | | | | | | | | |  |
|  |  |  |  |  | ወደጤናተቃምእንድሄድተደርጋል 99/ መልስየለም | | | | | | | | | | | | | | | | | | | | | | | | | | | | | | | | | | | | |  |

**ልትብብሮእጅግአመሰግናለሁ!!!!**
