# Supplementary material for: Uptake of complete postnatal care services and its determinants among rural women in Southern Ethiopia: Community-based cross-sectional study based on the current WHO recommendation
Source: PLoS One. 2021 Feb 3;16(2):e0246243. doi: 10.1371/journal.pone.0246243 (PMC7857562; doi:10.1371/journal.pone.0246243)
Supplement: Attachment — Submitted filename: Response to Reviewers.docx [file pone.0246243.s003.docx]

**AUTHORS’ RESPONSE TO EDITOR AND REVIEWERS**

I. **AUTHORS’ RESPONSE TO EDITOR**

**Dear: Onikepe Oluwadamilola Owolabi**

We thank you and the reviewers for a thorough reading and constructive criticism of our manuscript and for the opportunity to revise and resubmit. We are pleased to submit the revised research article “Uptake of Complete postnatal care services and its determinants among women in Ezha district, Guraghe zone, Southern Ethiopia, 2019: Community-based cross-sectional study based on the current WHO recommendation” for your consideration in the special collection of *PLOS ONE*. On the following pages, you will find our response to the editor’s comments. On behalf of my co-authors, I thank you for your consideration of this resubmission. We appreciate your time and look forward to your response.

Sincerely,

Aklilu H.(MPH)(corresponding author)

[**aklilihabte57@gmail.com**](mailto:aklilihabte57@gmail.com)

**General comments**

**Comment from The Review Process:** Thank you for submitting your manuscript to PLOS ONE. After careful consideration, we feel that it has merit but does not fully meet PLOS ONE’s publication criteria as it currently stands. Therefore, we invite you to submit a revised version of the manuscript that addresses the points raised during the review process.

**RESPONSE:** Thank you for coordinating the review process. We have revised the manuscript by exploiting all our maximum efforts and tried to address Reviewer’s comments in advance.

**Comment1:** Please ensure that your manuscript meets PLOS ONE's style requirements, including those for file naming. The PLOS ONE style templates can be found at <https://journals.plos.org/plosone/s/file?id=wjVg/PLOSOne_formatting_sample_main_body.pdf> and<https://journals.plos.org/plosone/s/file?id=ba62/PLOSOne_formatting_sample_title_authors_affiliations.pdf>

**Response**: After accessing those file templates we have checked and attest that all formatting style of the current manuscript and now we reformatted the manuscript accordingly and all these have been highlighted in the “revised manuscript with track changes’’

**Comment2**- Please try to be specific in your ethics statement whether participant consent was written or verbal. If verbal, please also specify: 1) whether the ethics committee approved the verbal consent procedure, 2) why written consent could not be obtained, and 3) how verbal consent was recorded.

**Response:** Thank you for your comment. Of course, the initial manuscript lacks detail explanation of the ethics statement. Now we have revised it and included ethical procedures that were not mentioned in the original manuscript in 3rd line of “Ethical approval and consent to participate” and this has been highlighted and underlined in the “revised manuscript with track changes” file. The ethics committee approved the verbal consent procedure.

**Comment3-** Please include additional information regarding the survey or questionnaire used in the study and ensure that you have provided sufficient details that others could replicate the analyses. For instance, if you developed a questionnaire as part of this study and it is not under a copyright more restrictive than CC-BY, please include a copy, in both the original language and English, as Supporting Information.

**Response:** We really appreciate for your critical comment. Actually we have mentioned about the how the tool was developed and employed for the study in the 2^nd^ line of “Data collection tools, methods and procedure”. It was developed by reviewing relevant literatures. Now we have incorporated both English and Amharic (original language) version of the questionnaire as supportive information, **“S1-Questionnaire”**

**Comment4-** Please amends your list of authors on the manuscript to ensure that each author is linked to an affiliation. Authors’ affiliations should reflect the institution where the work was done (if authors moved subsequently, you can also list the new affiliation stating “current affiliation:….” as necessary).

**Response:** Thank you for the comment. Now we have re-arranged affiliation of the authors with their current specific department and units where they are working. The amendments were based on the specific working unit of each author with their level of responsibility in the study. This has been highlighted in Pge1, line1of “revised manuscript with track changes” file.

__________________________________________________________________________________________________________END________________________________________

II. **AUTHOR’S RESPONSE TO REVIEWERS’ COMMENTS**

**Dear: Reviewers**

We thank you for a thorough reading and constructive criticism of our manuscript and for the opportunity to revise and resubmit. We are pleased to submit the improved research article “Uptake of Complete postnatal care services and its determinants among women in Ezha district, Guraghe zone, Southern Ethiopia, 2019: Community-based cross-sectional study based on the current WHO recommendation” for your consideration in the special collection of *PLOS ONE*. On the following pages, you will find our response to the reviewers’ comments. On behalf of my co-authors, I thank you for your consideration of this resubmission. We appreciate your time and look forward to your response independently as for reviewer#1 and reviewer#2 respectively.

Sincerely,

Aklilu H. (MPH)(corresponding author)

[**aklilihabte57@gmail.com**](mailto:aklilihabte57@gmail.com)

**AUTHOR’S RESPONSE TO REVIEWER**#**1**

**General comments**
**Comment1-**These issues are related to technical soundness of the manuscript and the conclusions part

**Response to reviewer1:** We really appreciate for your view. After your comments and suggestions we gave due emphasis in order to make the manuscript technically sound from its write up to detail methodology and analysis part. To assure representativeness, the sample size was determined for both first and second objective and we used the maximum sample size for the study. Regarding to the conclusion part, it’s based on our findings from the study without ignoring our initial objectives. But we kindly informing you that there were some statements which were omitted from the conclusion part of revised manuscript in order to avoid redundancy and those omitted narrations were already mentioned in the descriptive part of the result. This has been highlighted and underlined in the conclusion part of “revised manuscript with track changes” file.

**Comment2**- this issue is regarding to appropriateness of the statistical analysis been performed appropriately and rigorously?

Reviewer #1: NO

**Response:** We really appreciate your helpful examination of these errors. We are convinced with your concern, and the level of complete postnatal care is assessed in terms of those women who get at least one postnatal visit, in order to avoid overlooking of those mothers with no PNC. As reviewer 2 has also a similar concern, we have further clarified this in the result part and highlighted in the 2^nd^ to 3^rd^ line of “Level of complete post natal care utilization’’ of revised manuscript with track changes’’ file. Let us explain why this error has been committed. Initially we perceived that excluding those mothers without PNC from the analysis mayn’t cause significant difference in the level of CPNC because of their relatively small count. But now we are in agreement with your concern and corrected it accordingly throughout the current manuscript.

**Comment3-**Have the authors made all data underlying the findings in their manuscript fully available? **(Reviewer1= NO)**

**Response:** We appreciate the comment and we have uploaded our supplementary information including the full data set, English and Amharic (local language) version of the questionnaire and result of bivariate analysis in the current submission.

**Comment4-** Regarding presentation of the manuscript in an intelligible fashion and written in Standard English **(Reviewer1=Yes).**

Response: thank you for your constructive and positive view regarding the manuscript.

**Comment5-** The authors measured PNC based on the WHO definition which takes timing into consideration. How did the authors treat births at home? Were they dropped from the analysis? This needs to be mentioned in this section. It is also important to mention the type of personnel.

**Response:** We really appreciate for your view. Regarding home at births, they were not dropped from the analysis. Initially during conceiving of the study we were planned to put home births in exclusion criteria, but we let them to be part of the study for two reasons:

1. Currently, every Kebele of the district have adequate number of health extension workers to its population and once home delivery has been occured the health extension worker/s immediately accessed that woman within 24 hours of duration and she might have a probability of receiving the PNC services. This was a little bit supported by the study in which 2 mothers with home delivery got complete post natal care service.
2. The institutional review board also raised the issue of excluding those mothers with home birth from the study as unethical issue.

__________________________________________________________________________________________________________END________________________________________

**AUTHOR’S RESPONSE TO REVIEWER**#**2**

**General comments**
**Comment1-**These issues are related to technical soundness of the manuscript and the conclusions part (Reviewer2=YES)

**Response:** We really appreciate for your positive view.

**Comment2**- this issue is regarding to appropriateness of the statistical analysis been performed appropriately and rigorously?

Reviewer #2: No

**Response:** We really appreciate your helpful examination of these errors. Let us explain why this error has been committed. Initially we thought that excluding those mothers without PNC from the analysis mightn’t cause significant difference in the level of CPNC because of their relatively small count. We are convinced with your concern, that the level of complete postnatal care is assessed in terms of those women who get at least one postnatal visit, in order to avoid overlooking of those mothers with no PNC. As reviewer 1 have also a similar concern, we have further clarified this in the result part and highlighted in the “Level of complete post natal care utilization’’ of revised manuscript with track changes’’ file. But now we are in agreement with your concern and corrected it accordingly in the current manuscript.

**Comment3-**Have the authors made all data underlying the findings in their manuscript fully available? **(Reviewer2=Yes)**

**Response:** thank you for the positive comment.

**Comment4-** Regarding presentation of the manuscript in an intelligible fashion and written in Standard English.

**Response**: thank you for your constructive comment. First we are in agreement with your view and we gave due emphasis and endeavor to solve the issue throughout the manuscript.

**Comment5-**Title of the article complete postnatal care utilizations and its associated factors among women who gave birth in the last 12 months in Ezha district, Southern Ethiopia, 2019: Community- based cross-sectional study: Compliance to the current WHO recommendation. The subject is relevant and brings current and important information to the context of maternal and neonatal health in Africa. The findings are substantial and may contribute to health policies implementation in low- and middle-income settings. However, the text needs several improvements in order to grant proper discussion and analysis of included issues.

**Response:** We would like to thank the reviewer for your kind words and supportive ideas about the current study as it is a contemporary issue. We had given a due emphasis to make improvements in each and every section of the manuscript accordingly.

**Comment6-**The English of the manuscript needs significant proofreading.

**Response:** We have revised as per your recommendations and we tried to manage those issues in-depth. We have tried to show most of these amendments in the “revised manuscript with track changes”

**Comment7**-The importance of complete postnatal care utilization is not well emphasized. As you mentioned on page 3 and 3rd paragraph, “within the first week of life increases…”. What’s the rationale to compare the complete PNC? Where all other PNC utilized are categorized as sporadic/non-case?

**Response7.1:** We really appreciate your helpful examination. There were shortage of literatures that directly written on complete postnatal care. But we are trying to address the importance of CPNC in terms adequate PNC visit schedules and were mentioned in Page4Paragraph1, 1^st^ -5^th^ lines and last paragraph of ‘Introduction’ part of revised manuscript.

**Response7.2:** “within the first week of life increases…”. Now this is corrected as “within the first six weeks” in page, 3 line23 of revised manuscript.

**Response7.3:** Thank you for your concern. When we come to our reason for comparison, every visit schedule has its own importance in provision of contents of care. For example last visit schedule at sixth week is important for the woman and newborn in initiating for vaccination, family planning and health educations regarding to essential nutrition actions. Now we are comparing provision of contents of care between mothers with complete and sporadic PNC and we appreciate huge differences in between. So, this might be clearly showed that importance of receiving CPNC to get adequate contents of care, this in turn support the importance of the current study.

**Comment8-** “On the data analysis section “continuum of care was mentioned”?

Response8: sorry for the inconvenience that’s wrongly written and now it is corrected as “CPNC” in 24^th^ line of “data analysis” in “revised manuscript with track changes”.

**Comment9-** Page 13 on the prevalence of complete postnatal care utilization section- those mothers who never sought PNC were overlooked and presented. I was wondering if mothers with home delivery were in your exclusion criteria. Also, considering the national facility delivery proportion, the number of mothers who sought 1st PNC service is too high according to your findings. Could you comment that on the manuscript?

**Comment9.1:** Page 13 on the prevalence of complete postnatal care utilization section- those mothers who never sought PNC were overlooked and presented.

**Response9.1:** we are greatly appreciating your comment. We had tried to address this issue in response 2 of your comment2. Initially, we thought that excluding those mothers without PNC from the analysis mightn’t cause significant difference in the level of CPNC because of their relatively small count. But now, we are in agreement with your concern, that the level of complete postnatal care is assessed in terms of those women who get at least one postnatal visit, in order to avoid overlooking of those mothers with no PNC. As reviewer 1 have also a similar concern, we have further clarified this in the result part and highlighted in the 1^st^ to 3^rd^ line of “Level of complete post natal care utilization’’ of revised manuscript with track changes’’ file.

**Comment9.2-**I was wondering if mothers with home delivery were in your exclusion criteria.

**Response9.2:** We really appreciate for your view. Regarding home deliveries, they were part of the study and not dropped from the analysis. Initially during conceiving of the study we were planned to put home births in exclusion criteria, but we let them to be part of the study in two reasons:

1. Currently, every Kebele of the district have adequate number of health extension workers to its population and once home delivery has been happened the health extension worker/s immediately accessed that woman within 24 hours of duration and she might have a probability of receiving the PNC services. This was a little bit supported by the study in which 2 mothers with home delivery got complete post natal care service.
2. The institutional review board also raised the issue of excluding those mothers with home birth from the study as unethical issue.

**Comment9.3-**Also, considering the national facility delivery proportion, the number of mothers who sought 1st PNC service is too high according to your findings. Could you comment that on the manuscript?

**Response9.3:** in comparison with report of EDHS2016 and mini-EDHS2019 report, all maternal health services in the study area were with higher coverage. We also noticed that high PNC1 in the current study, this might be due to high proportion skilled delivery service in the study area.

**Comment10-** On the outcome variable – Sporadic vs complete postnatal care- mothers who don’t get postnatal care are overlooked in this research.

**Response10:** we are greatly appreciating your comment. We had tried to address this issue in **response 9.1** of your **comment9**. Initially, we thought that excluding those mothers without PNC from the analysis mightn’t cause significant difference in the level of CPNC because of their relatively small count. But now, we are in agreement with your concern, that the level of complete postnatal care is assessed by taking of those mothers who get at least one postnatal visit, in order to avoid overlooking of those mothers with no PNC. As reviewer 1 have also a similar concern, we have further clarified this in the result part and highlighted in the 1^st^ to 3^rd^ line of “Level of complete post natal care utilization’’ of revised manuscript with track changes’’ file.

**Comment11-** Was PNC considered after discharge for CS? Or while women were admitted?

**Response11:** in this study all visits other than recommended PNC schedules (like maternal and newborn illness including C/S) were not considered as PNC service.

**Comment12-** the wealth status categories consider poorest to richest instead of lowest to highest

**Response12:** this was corrected accordingly and highlighted in the result part page14, table 1 of” revised manuscript with track changes” file.

**Comment13-** Include more detailed limitations of the study. Causal relationship is impossible in this study design, not difficult. What type of recall bias is likely? What women were missing from the study, e.g. Is it possible that the CHEW birth registers missed women and these women would be the most vulnerable and least likely to get PNC?

**Comment13.1-** Include more detailed limitations of the study.

**Response13.1:** Thank you for your comment. We are included all the possible limitation of the study in the current manuscript as per your recommendation in line 12-16^th^ of page24 of “revised manuscript with track changes”. There might be a possibility of recall bias since women were asked for events which have already happened within the past one year prior to this study.

**Comment13.2-** What women were missing from the study, e.g. Is it possible that the CHEW birth registers missed women and these women would be the most vulnerable and least likely to get PNC?

**Response13.2:** we got it as basic concern raised. The district was implementing community health information system (CHIS), in which every house hold of the district had a family folder that is updated on monthly basis. Every vital event in each kebele was recorded on timely basis since home –to- home visit has been conducted by HEWs. So, missing those women who delivered in the last 12 months from registration could be unlikely.

**Comment14-** What about women with stillbirths or early neonatal deaths? were they included? What difference would it make?

**Response14:** we are greatly appreciating your comment. Those women with still birth and early neonatal death were included in our study. Since most of contents of care provided during postpartum visits are maternal part and those mothers have to get PNC services as support tailored to address acute postpartum issues and each woman’s individual needs and it is better to make them part of the current study.

**Comment15-** The discussion is full of results already displayed and lacks depth in addressing the actual findings. In addition to quoting and comparing previous studies with current results, reflections on present findings should be conducted, e.g. addressing gender issues. The ideas are disclosed, however could be further elaborated in order to improve the manuscript's strength.

**Response15:** Thank you very much for your in-depth review and comment regarding the discussion part. We are incorporating all your comments and suggestions in each and every paragraph from the beginning to the end and all those changes are highlighted in the “revised manuscript with track changes”. In addition, some references were added in the current manuscript and those are highlighted in the revised manuscript with track changes by mentioning as’…new reference”

**Comment16-** PLOS authors have the option to publish the peer review history of their article ([what does this mean?](https://journals.plos.org/plosone/s/editorial-and-peer-review-process#loc-peer-review-history)). If published, this will include your full peer review and any attached files. If you choose “no”, your identity will remain anonymous but your review may still be made public.
**Do you want your identity to be public for this peer review?** For information about this choice, including consent withdrawal, please see our [Privacy Policy](https://www.plos.org/privacy-policy).

Reviewer #2: No

**Response**: Yes

__________________________________________________________________________________________________________END________________________________________
