# Supplementary material for: Uptake of complete postnatal care services and its determinants among rural women in Southern Ethiopia: Community-based cross-sectional study based on the current WHO recommendation
Source: PLoS One. 2021 Feb 3;16(2):e0246243. doi: 10.1371/journal.pone.0246243 (PMC7857562; doi:10.1371/journal.pone.0246243)
Supplement: Attachment — Submitted filename: Response to Reviewers.docx [file pone.0246243.s004.docx]

**AUTHORS’ RESPONSE TO ACADEMIC EDITOR AND REVIEWERS**

**Dear: Onikepe Oluwadamilola Owolabi, Academic Editor**

We thank you and the reviewers for a thorough reading and constructive criticism of our manuscript and for the opportunity to revise and resubmit. We are pleased to submit the revised research article “Uptake of Complete postnatal care services and its determinants among rural women in Southern Ethiopia: Community-based cross-sectional study based on the current WHO recommendation” for your consideration in the special collection of *PLOS ONE*. On the following pages, you will find our response to the editor’s comments. On behalf of my co-authors, I thank you for your consideration of this resubmission. We appreciate your time and look forward to your response.

Sincerely,

Aklilu H.(MPH)(corresponding author)

[**aklilihabte57@gmail.com**](mailto:aklilihabte57@gmail.com)

**General comments**

**Comment from The Review Process:** Thank you for submitting your manuscript to PLOS ONE. After careful consideration, we feel that it has merit but does not fully meet PLOS ONE’s publication criteria as it currently stands. Therefore, we invite you to submit a revised version of the manuscript that addresses the points raised during the review process.

**RESPONSE:** Thank you for coordinating this review process. We reviewed the manuscript taking full advantage of our efforts and attempted to respond to the reviewer's comments in advance.

**Comment:** Please submit your revised manuscript by Jan 01 2021 11:59 PM. If you will need more time than this to complete your revisions, please reply to this message or contact the journal office at plosone@plos.org. When you're ready to submit your revision, log on to https://www.editorialmanager.com/pone/ and select the 'Submissions Needing Revision' folder to locate your manuscript file.

**Response:** Thank you for taking the time to submit the revised manuscript to us on time and we will attempt to submit the revised manuscript within the required timeframe.

**Comment:** If you would like to make changes to your financial disclosure, please include your updated statement in your cover letter. Guidelines for resubmitting your figure files are available below the reviewer comments at the end of this letter.

**Response:** Thank you for the suggestion, but there are no changes to financial disclosure ( i.e no funding to report)

**Comment:** If applicable, we recommend that you deposit your laboratory protocols in protocols.io to enhance the reproducibility of your results. Protocols.io assigns your protocol its identifier (DOI) so that it can be cited independently in the future. For instructions see: <http://journals.plos.org/plosone/s/submission-guidelines#loc-laboratory-protocols>

**Response:** This search was not based on laboratories and has no laboratory protocol for depositing here.

__________________________________________________________________________________________________________END________________________________________

THANK YOU!!!

I. **AUTHOR’S RESPONSE TO REVIEWERS’ COMMENTS**

**Dear: Reviewers**

We thank you for a thorough reading and constructive criticism of our manuscript and for the opportunity to revise and resubmit. We are pleased to submit the improved research article “Uptake of Complete postnatal care services and its determinants among rural women in Southern Ethiopia: Community-based cross-sectional study based on the current WHO recommendation” for your consideration in the special collection of *PLOS ONE*. On the following pages, you will find our response to the reviewers’ comments. On behalf of my co-authors, I thank you for your consideration of this resubmission. We appreciate your time and look forward to your response independently as for reviewer#3.

Sincerely,

Aklilu H. (MPH)(corresponding author)

[**aklilihabte57@gmail.com**](mailto:aklilihabte57@gmail.com)

**AUTHOR’S RESPONSE TO REVIEWER**#**3**

**Comments to the Author**

**Comment1**: If the authors have adequately addressed your comments raised in a previous round of review and you feel that this manuscript is now acceptable for publication, you may indicate that here to bypass the “Comments to the Author” section, enter your conflict of interest statement in the “Confidential to Editor” section, and submit your "Accept" recommendation.

Reviewer #3: (No Response)

**Response:** First, we would like to hear from you and your suggestion. We have tried to address all of the potential comments and suggestions raised by reviewers 1 and 2 in the previous review process and now we are also encouraged to consider all of your suggestions and comments.

**Comment2:** Is the manuscript technically sound, and do the data support the conclusions?

The manuscript must describe a technically sound piece of scientific research with data that supports the conclusions. Experiments must have been conducted rigorously, with appropriate controls, replication, and sample sizes. The conclusions must be drawn appropriately based on the data presented.

Reviewer #3: Partly

**Response:** We appreciate what you have to say. After your comments and suggestions, we have emphasized making the manuscript technically sound from its writing to the detailed methodology and analysis portion. To ensure representativeness, the sample size was determined for the first and second objectives and the maximum sample size was used for the study. Concerning the conclusion, it is based on the findings of the study without ignoring our initial objectives.

**Comment3:** Has the statistical analysis been performed appropriately and rigorously?

Reviewer #3: Yes

**Response:** We thank you for your constructive and positive viewpoint regarding the analysis portion of the study.

**Comment4:** Have the authors made all data underlying the findings in their manuscript fully available?

The PLOS Data policy requires authors to make all data underlying the findings described in their manuscript fully available without restriction, with rare exceptions (please refer to the Data Availability Statement in the manuscript PDF file). The data should be provided as part of the manuscript or its supporting information, or deposited in a public repository. For example, in addition to summary statistics, the data points behind means, medians, and variance measures should be available. If there are restrictions on publicly sharing data—e.g. participant privacy or use of data from a third party—those must be specified.

Reviewer #3: Yes

**Response:** We appreciate your assurance regarding the availability of all necessary data.

**Comment5:** Is the manuscript presented in an intelligible fashion and written in standard English?

PLOS ONE does not copyedit accepted manuscripts, so the language in submitted articles must be clear, correct, and unambiguous. Any typographical or grammatical errors should be corrected at revision, so please note any specific errors here.

Reviewer #3: No

**Response:** We greatly appreciate your efforts to thoroughly review the manuscript to detect typographical or grammatical errors. In the present revision of the manuscript, we have placed a strong emphasis on this part and attempt to correct all possible grammatical and typological errors in detail. All necessary changes and corrections in each section of the manuscript have been indicated in the “revised manuscript with track changes” file.

**6. Review Comments to the Author**

**General comment of reviewer 3:** The manuscript highlights an important issue of maternal health. However, the soundness of the methodology needs to be improved.

Response: First of all, we would appreciate your constructive input. Regarding the soundness of the methodology, we are trying to explore the whole methodology section of the manuscript after your comment and we will try to make it sounder. All possible revisions in the “Data Collection Tools and Personnel”, “Data Quality”, and “Data Analysis” sections were highlighted in the “Revised Manuscript with Change Tracking” file.

**Major revision**

**Comment1:** The title is too long; it may make confuse the reader. The year can be indicated in the methods section instead of in the title.

**Response**: Thank you for your pertinent comment to reduce the confusion concerning the title and now it is corrected as “Uptake of Complete postnatal care services and its determinants among rural women in Southern Ethiopia: Community-based cross-sectional study based on current WHO recommendation”

**Comments in the Method part**

**Comment:** The estimation of the study population size should be given since the study aimed to assess the coverage of PNC uptake.

**Response:** Thank you for your concern about determining the sample size. We calculate the sample size in two ways; for the first and second objectives, as we try to clarify that in the 1^st^ to 10^th^ line of the “sample size determination” portion of the “Manuscript file”. Subsequently, the maximum sample size obtained from the second objective (i.e. for the CPNC determinants) was used for the study. All of this was done according to our advisors during the proposal development phase.

**Comment:** The sentence on page 6 of the sampling techniques, “The district consists of 28 Kebeles” and “Kebele is the smallest administrative unit in Ethiopia” are redundant since there is similar information under the study setting.

**Response:** thank you for an in-depth view and now it is omitted and highlighted in the “sampling technique” section of the “revised manuscript with track changes” file

**Comment:** Was there a sampling frame of households in each Kebeles? Please elaborate.

**Response:** Firstly, we would like you to ask us to respond to this sampling question as part of the research process. Initially, we obtained the list of women who gave birth within the last 12 months and who remained for six weeks or more after delivery from the registry of each health post, because each delivery was recorded daily. After verifying the presence of these eligible women, codes/numbers were provided for the homes, and a sampling frame was developed for each Kebele. All of these procedures were highlighted in the “Sampling Technique” section of the “Revised manuscript with Track Changes” file, lines 6-11.

**Comment:** The authors wrote, “A pre-tested structured questionnaire was developed by reviewing relevant literature with reasonable modifications [21-23, 36, 37].” Could you explain the rationale to use those questionnaires? Please also explain.

**Response:** Just to show that the tool/questionnaire we used to collect the data was developed by looking at different pieces of literature in the area of interest. The references mentioned have already been the subject of postnatal care research. The word “Pre-tested” is indicated to show that the questionnaire was tested prior to the start of the research and is well elaborated in the “Data Quality Management” section of the “Revised Manuscript with Track Changes”, page8, lines 5-8.

**Comment:** The theoretical framework that the researchers used for the development of the research instruments.

**Response:** We appreciate the question. As we attempt to respond to the above comment, we developed the research instrument after reviewing various research carried out in postnatal care and more specifically on complete postnatal care.

**Comment:** Definition and operationalization of variables of the study should be placed before the data analysis.

**Response:** We accept your recommendation and do so accordingly.it is highlighted in the ‘revised manuscript with track change’, Page 8.

**Comments in the result part**

**Comment:** Figure 3, why did the authors exclude women with no PNC from the analysis? I think it should be included in the analysis.

**Response:** We have taken this as a mistake and we are doing so based on your recommendation. We also omitted this figure in the current revised manuscript since it is already mentioned in the form of a declaration.

**Comment to the discussion section.**

**Comment:** The discussion is lacking the elaboration on public health/health service implications of the findings. What are the lessons from this study for other contexts of low and middle-income countries?

**Response:** Thank you very much for this comment, and we have received these suggestions and recommendations as a necessary contribution to strengthen the implications of our findings. We try to underline what the results mean and what measures should be taken by the stakeholders based on the findings and all these are mentioned and highlighted in the 'discussion' part of the 'manuscript with track changes' file.

**Comment**: The discussion is lacking in the description of the limitations and strength of the study.

**Response:** thank you for the comment. We understood that it was our fault, in a manuscript submitted earlier, to clearly show the strength and limitations of our study. But now we have tried to clarify these limitations and strengths accordingly and have highlighted them in the last paragraph of the 'discussion portion' of the 'revised manuscript with track changes'	file, Page22

**Minor revision**

**Comment:** The manuscript contains many typos. The use of capital letters is often improper. Please do the re-editing.

**Response:** We would like to appreciate you for an in-depth examination of our manuscript. We revised the whole document according to your recommendations, line by line, and we tried to manage these problems. We tried to show all of these changes in the “revised manuscript with track changes”.
**Comment:** Mean and standard deviation should be written as a number after the point (write 28.8 +5.30 instead of 28.84 + 5.306 (page 10)

**Response:** this error occurred in the 'Socio-demographic Characteristics of Respondents' portion of the 'result' part and is now corrected.

**Comment:** Make the figure as two dimensions one, instead of three dimensions.

**Response:** Thank you very much for your meticulous observation to our work. But initially, we convert these figures using Preflight Analysis Conversion Engine (PACE) per the PLOS ONE manuscript submission guideline. If other options are available, we are welcome to do so.

**Comment:** Pie charts and bar charts are not preferable for presenting the results since you already have the tables with similar content.

Response: thank you for your valuable comment. According to your suggestion, we have omitted the figures which lead us to redundancy.

**Comment:** PLOS authors have the option to publish the peer review history of their article (what does this mean?). If published, this will include your full peer review and any attached files. If you choose “no”, your identity will remain anonymous but your review may still be made public.

**Response:** No

Comment: Do you want your identity to be public for this peer review? For information about this choice, including consent withdrawal, please see our Privacy Policy.

Reviewer #3: No

**Response:** no

__________________________________________________________________________________________________________END________________________________________

**With kind regards!!**
