# Supplementary material for: Uptake of complete postnatal care services and its determinants among rural women in Southern Ethiopia: Community-based cross-sectional study based on the current WHO recommendation
Source: PLoS One. 2021 Feb 3;16(2):e0246243. doi: 10.1371/journal.pone.0246243 (PMC7857562; doi:10.1371/journal.pone.0246243)
Supplement: Attachment — Submitted filename: response to reviewer.docx [file pone.0246243.s006.docx]

**AUTHORS’ RESPONSE TO ACADEMIC EDITOR**

**Dear: Frank T. Spradley, Academic Editor**

We thank you and the reviewers for a thorough reading and constructive criticism of our manuscript and for the opportunity to revise and resubmit. We are pleased to submit the revised research article “Uptake of Complete postnatal care services and its determinants among rural women in Southern Ethiopia: Community-based cross-sectional study based on the current WHO recommendation” for your consideration in the special collection of *PLOS ONE*. On the following pages, you will find our response to the editor’s comments. On behalf of my co-authors, I thank you for your consideration of this resubmission. We appreciate your time and look forward to your response.

Sincerely,

Aklilu H.(MPH)(corresponding author)

**Comments From Academic Editor**
PONE-D-20-05045R3
Uptake of Complete postnatal care services and its determinants among rural women in  Southern Ethiopia : Community-based cross-sectional study based on the current WHO recommendation
PLOS ONE

Dear Dr. Habte,

Thank you for submitting your manuscript to PLOS ONE. After careful consideration, we feel that it has merit but does not fully meet PLOS ONE’s publication criteria as it currently stands. Therefore, we invite you to submit a revised version of the manuscript that addresses the points raised during the review process.
**Comment:** The manuscript has been revised according to the reviewers' comments. However, one noticeable typographical error remains: in table 4, it should read "body weight" not "bodyweigh". Please correct.

**Response:** We appreciate your meticulous review of our work. In the revised manuscript, we corrected it and highlighted it in Table 4, page 14 of the "Revised Manuscript with Track Changes "

Please submit your revised manuscript by Mar 01 2021 11:59PM. If you will need more time than this to complete your revisions, please reply to this message or contact the journal office at [plosone@plos.org](mailto:plosone@plos.org). When you're ready to submit your revision, log on to <https://www.editorialmanager.com/pone/> and select the 'Submissions Needing Revision' folder to locate your manuscript file.

Please include the following items when submitting your revised manuscript:

- A rebuttal letter that responds to each point raised by the academic editor and reviewer(s). You should upload this letter as a separate file labeled 'Response to Reviewers'.
- A marked-up copy of your manuscript that highlights changes made to the original version. You should upload this as a separate file labeled 'Revised Manuscript with Track Changes'.
- An unmarked version of your revised paper without tracked changes. You should upload this as a separate file labeled 'Manuscript'.

If you would like to make changes to your financial disclosure, please include your updated statement in your cover letter. Guidelines for resubmitting your figure files are available below the reviewer comments at the end of this letter.

If applicable, we recommend that you deposit your laboratory protocols in [protocols.io](http://protocols.io/) to enhance the reproducibility of your results. Protocols.io assigns your protocol its own identifier (DOI) so that it can be cited independently in the future. For instructions see: <http://journals.plos.org/plosone/s/submission-guidelines#loc-laboratory-protocols>

We look forward to receiving your revised manuscript.

Kind regards,

Frank T. Spradley
Academic Editor
PLOS ONE

_________________________________________________________________________________________________________END________________________________________

THANK YOU!!!
